# Macroporous Monolithic Polymers: Preparation and Applications

**DOI:** 10.3390/ma2042429

**Published:** 2009-12-18

**Authors:** Ruben Dario Arrua, Miriam Cristina Strumia, Cecilia Inés Alvarez Igarzabal

**Affiliations:** Haya de la Torre y Medina Allende, Edificio de Ciencias II, Departamento de Química Orgánica, Facultad de Ciencias Químicas, Universidad Nacional de Córdoba, Ciudad Universitaria (5000) Córdoba, Argentina; E-Mails: darrua@mail.fcq.unc.edu.ar (R.D.A.); mcs@mail.fcq.unc.edu.ar (M.C.S.)

**Keywords:** macroporous monoliths, macroporous polymers, chemical modification

## Abstract

In the last years, macroporous monolithic materials have been introduced as a new and useful generation of polymers used in different fields. These polymers may be prepared in a simple way from a homogenous mixture into a mold and contain large interconnected pores or channels allowing for high flow rates at moderate pressures. Due to their porous characteristics, they could be used in different processes, such as stationary phases for different types of chromatography, high-throughput bioreactors and in microfluidic chip applications. This review reports the contributions of several groups working in the preparation of different macroporous monoliths and their modification by immobilization of specific ligands on the products for specific purposes.

## 1. Introduction

The recent advances in solid phase synthesis, catalysis or different types of separation have raised great interest in the area of cross-linked polymeric supports at both the academic and industrial level. Introduced fifty years ago, this type of polymers was initially produced in the form of particles [1] and is currently used for filling columns in processes that are carried out under continuous flow, as in different chromatography processes. Several applications of these supports involve polymers with low cross-linked degree (expandible or homogeneous polymers), which require a previous swelling process to became porous. When these polymers swell in a good solvent, they allow the access of all reagents to all material sites although their use becomes limited. On the other hand, the changes in the type of solvent used usually damage the properties of the material.

Contrary to the fact that the homogeneous polymeric networks need to swell to reach a porous state, the macroporous polymers (heterogeneous network) has a porous structure formed during preparation and maintained in any solvent or in dry state. The inner structure consists of aggregates of microglobules of interconnected polymers forming pores and their rigidity results from their high degree of cross-linking.

Generally, these materials are prepared from a suspension polymerization reaction in particle form [2], in which the polymerization mixture should always include a vinylic monomer as cross-linker (it must have at least two double bonds) and an inert solvent or a mix of inert solvents (called porogen, porogenic mixture or pore forming agent). The presence of the inert solvent is crucial for the preparation of macroporous polymers. Hence, different classes of solvents are used: those that solvate the polymeric chains in formation (good solvents) [3]; those that do not solvate the polymeric chains in formation (bad solvents) [3]; supercritic carbon dioxide [4,5]; lineal polymers [6]; or a mix of good/bad solvents [7].

In the last years, macroporous monolithic materials in rod form have been introduced as a new and useful generation of macroporous polymers prepared in a more simple way respect to suspension, from a homogenous mixture formed by vinylic/divinylic monomers and the inert or a mix of inert solvents, into a mold. The main objective of this review is to report important contributions of several groups working in the preparation of these macroporous monoliths and their modification for specific purposes.

### 1.1. Mass Transport in Processes That Use Polymers in Particle Form

At present, porous particles with different size, porosity and chemical composition are used as column packing and applied to different processes, such as affinity chromatography. This technique involves the separation, isolation and/or purification of biological compounds and is based on biospecific interactions between the biomolecule to be purified and a ligand, which is bound to a polymeric matrix.

This type of support presents advantages in chromatography, in spite of two inherent limitations: the great interparticle volume, and the slow diffusion of high-molecular-weight solutes into the pores of the particles [8]. Concerning the latter, the movement or flow of the solutes (dissolved in a solvent) into the pores is due to their capacity to diffuse. While solutes of low molecular weight are capable of moving relatively fast, solutes of high molecular weight (proteins, polysaccharides or synthetic polymers) move more slowly, since they present lower diffusion coefficients. Thus, this problem becomes important in processes in which the rate of mass transfer is the determining factor in chromatographic separations, catalysis, *etc.* [9].

The resistance of large molecules to mass transfer is mainly due to the discontinuity found in packed columns with particulate system due to the empty spaces between the particles. Theoretical studies reveal that the void volume corresponding to the empty spaces between the particles (considering spherical particles of the same size) as a result of the particulate character of the packing represents at least 30–40% of the total volume of the column. Therefore, the mobile phase flows mostly through the interstitial voids between the particles in which the resistance at the flux is less. On the other hand, the liquid in the pores practically does not flux, remaining stagnated [8,9,10]. When the molecules of the solute are in the mobile phase, they begin to migrate mainly in the inter particle voids. Then, as a consequence of the difference in concentration between the solute in the mobile phase and the liquid in the pores, the molecules begin to diffuse toward the interior of the pores of the particles until reaching the concentration equilibrium between them. Then, the concentration of the solutes in the mobile phase decreases, the gradient in concentration is reversed, and the molecules of the solute flux, in this case from the interior of the pores toward the liquid that fluxes in the inter particle spaces [8,9].

In the case of low-molecular-weight solutes (small organic molecules and ions), the molecules diffuse fast and the concentration equilibrium in the pores is quickly reached. Nevertheless, in the case of high-molecular-weight solutes (such as proteins, nucleic acids and synthetic polymers), the macromolecule diffuses slowly toward the interior of the pores, interacts with the polymer active site, and then diffuses toward the mobile phase that fluxes in the inter particle spaces. Now then, if the velocity of diffusion is smaller than the time necessary for the interaction between the biological macromolecule and the ligand bound to the support to occur, the first is the determiningt step in the process. In these cases, the molecules of solute interact only with the few and more accessible active sites into the pores, producing a less effectiveness in the separation medium. By all reasons previously mentioned, large columns or slow fluxes must be used [9].

### 1.2. Increase in the Mass Transfer by Convection

The use of solid catalysts with large pores has shown that mass transfer is enhanced when it occurs by convection [11]. Here, the mobile phase flows to increase the mass transfer of solutes. Therefore, polymeric supports with large pores would be necessary for high-molecular-weight solutes to be transported by convection. However, most polymers in particle form present pores up to 100 nm, not being adequate to achieve a convective transport.

**Figure 1:**
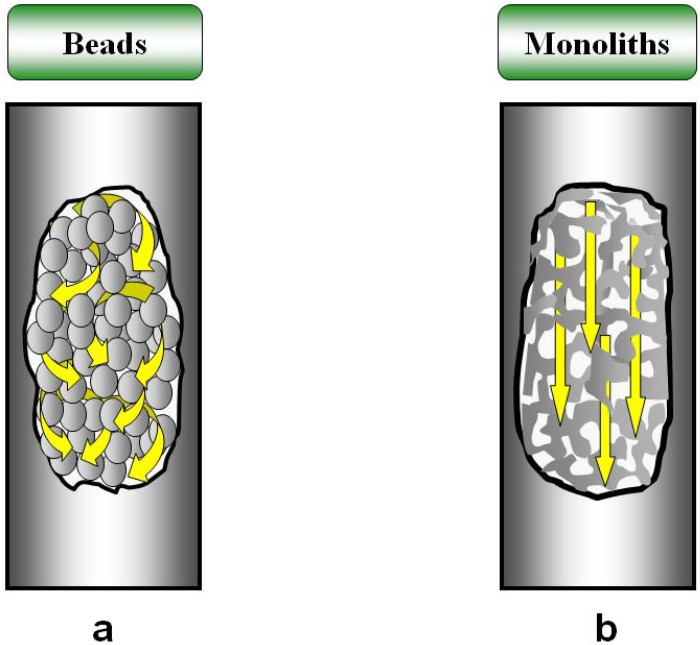
Passage of the mobile phase through particulate media (macroporous particles) (a) and continuous support (macroporous continuous rods) (b).

Therefore, particulate media with larger pores began to develop [9]. Porous poly(styrene-co-divinylbenzene) [poly(Sty-co-DVB)] particles with pores of approximately 400 nm were used for separation of biopolymers. Even with this porous size, the mobile phase flows mainly between the inter particle voids since the transport by convection in the above example [9,12] is only 2% of the total; yet this low percentage was sufficient to improve the separation of biopolymers. Theoretical studies indicate that the maximum effect by convective transport could be achieved if the mobile phase is forced to go through the porous medium [13]. As mentioned above, particulate systems always have an important inter particle volume through which the mobile phase flows preferentially. Thus, new materials such as “continuous” macroporous polymeric systems were designed to reduce or avoid discontinuity and to solve the problem of flow through inter particle spaces. The major advantage of these new “monolithic materials” has been the increase in the mass transfer by convective transport, since in this case the mobile phase is forced to cross the whole separation medium [10]. Therefore, resolution, efficiency and the dynamic binding capacity are independent of flow rate. This variation in the passage of the mobile phase is shown in Figure 1.

Monolithic columns create a “single large particle” that fills the column entirely, allowing no void. Within this monolith, a series of connected pores forms a continuous skeleton, filled with interconnected pores that form flow channels of a consistent size [14]. The monolithic column developes a network of channels in the continuous phase of a porous material that shows high axial permeability, a large internal pore surface area and less back pressure than that of conventional packed columns. Therefore, these monoliths allow performing separation processes at high flow rates and low back-pressures. The channels allow better contact between the analyte and the active sites of the stationary phase [8,10,14]. In addition, differences are found in the hydrodynamic properties. Whereas the pores are only partially used and diffusion is the major limitation in particle columns, the interphase mass transfer in monoliths is governed by convection, and the total pore volume is used.

Monoliths contain micrometer-sized pores responsible for convective flow, and smaller pores in the nanometer size range representing an important factor for the sorption capacity for small solutes in the total surface area.

Monolithic compressed soft gels based on poly(acrylamide) [polyAAm] were developed in 1989 by Hjerten to separate proteins [15]. Then, monolithic materials were prepared by Tennikova and Svec [16] from organic monomers, in a tubular mold. The polymeric materials were removed from the molds and sliced to obtain disks, which were placed in a cartridge and used to separate high-molecular-weight substances.

Furthering research, Svec and Frechet [17] developed monolithic materials in a column format. These monoliths were prepared by *in situ* polymerization into the chromatographic tube. On the other hand, the use of sol-gel chemistry to develop porous silica monoliths was introduced by Tanaka [18,19] and Siouffi [20]. Now then, the processes developed to improve the characteristics and performance of monolithic materials has developed into versatile and efficient alternatives to polymeric beads for a wide range of applications. Nowadays, monoliths can be formed *in situ* into any geometry (disks, rods and capillary columns) and advantageously made in sizes ranging from several liters [21] to a few nanoliters in the channel of a microfluidic chip [10].

Guiochon [22] and Unger *et al.* [23] reviewed the application of monolithic columns in high-performance liquid chromatography (HPLC) and compared the particle-packed and monolithic columns. Guiochon, however, further described the methods of comparison in the performance between monolithic and packed columns. On the other hand, Svec *et al.* [8,24,25,26] and Zou [27] reported the advances in the development of monolithic stationary phases, focusing on microscale chromatographic separations. Besides, in two important review articles, Svec reported the facets of the use of monoliths in preconcentration and solid-phase extraction [28] and in capillaries designed for separations in gas chromatography [29].

## 2. Continuous Macroporous Rods

**Figure 2:**
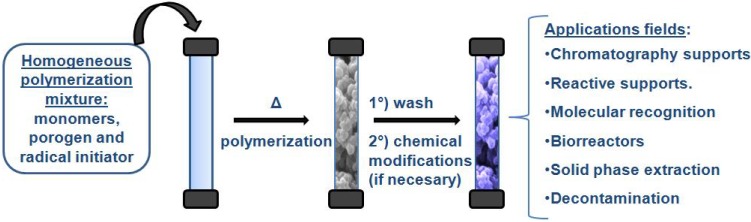
Different steps for the preparation of continuous polymer rods.

These materials include all the advantages of porous particulate systems: they are rigid and maintain their porous structure regardless of the solvent, even when dry. However, whereas porous particles are prepared by copolymerization reactions in suspension, these supports are synthesized in a very simple way from a homogeneous polymerization mixture, which contains the appropriate monomers [monovinyl monomer(s) and poly functional monomer(s) as cross-linking agent(s)], the radical initiator, and a set of porogenic solvents with suitable Θ value relative to the polymer formed [3,6,9,30,31]. The polymerization reaction takes place in a mold (which determines the form of the support) without agitation. Figure 2 shows an outline of the steps in the preparation of continuous macroporous polymer rods.

Once polymerized in a mold, the functional groups presented on the monolithic surface will depend of the monomer/s used. Then, these groups are used for the immobilization of biological catalysts or specific ligands as separation media by affinity chromatography through HPLC of small and large molecules.

**Figure 3:**
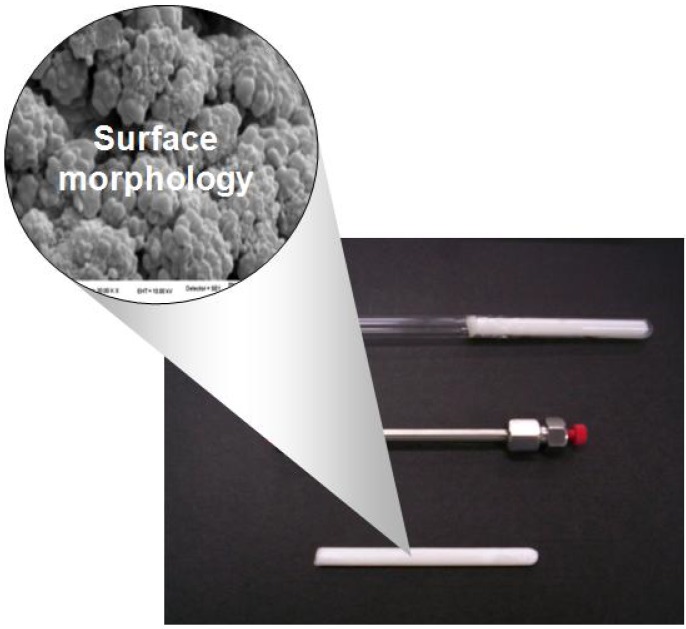
Macroporous monolithic rods prepared in a glass tube and a column of stainless steel HPLC.

For the synthesis of continuous porous polymer rods, the mold is generally a glass tube filled with the polymerization mixture. The tube is covered and then introduced into the container (at a given temperature) in which the polymerization reaction will occur. Usually, the polymerization reaction begins by thermal initiators which decompose and form free radicals so that the reactions run at a certain temperature (50–80 °C) [3,6,31,33,34,35,36,37,38]. Other syntheses are initiated by redox reactions [39] and also by UV radiation [25,40,41,42]. In the latter case, tubes of glass or fused silica capillaries are used as molds that do not absorb UV radiation. Additionally, other methods including radiation under ^60^Co γ-source [43,44,45] or by atom transfer radical polymerization [46] have been used in the synthesis of macroporous cross-linked organic polymeric monoliths. Tubes of different size and material such as stainless steel [3,37,38], poly(ether-ether-ketone) (PEEK) [47], glass [36] and silica capillaries [40] were also used as molds. Figure 3 shows different molds for the preparation of macroporous monolithic rods. As mentioned above, the continuous porous polymer synthesis is versatile and can be carried out under different experimental conditions.

## 3. Classical Mechanism of Pore Formation in Macroporous Polymers

The typical accepted mechanism for the formation of pores during common polymerization reactions in the presence of an inert solvent (precipitant) is as follows [48]: the organic phase contains the monomers, the porogenic agent and the radical initiator which decomposes at a certain temperature initiating the polymerization process "in solution". The polymer chains formed in solution precipitate as soon as they become insoluble in the reaction medium, either as a result of the cross-linking density of the polymer network, or depending on the porogenic agent used (thermodynamically "poor" solvent for the polymer). In this process, the monomers are thermodynamically better solvating agents for the polymer chains than the porogenic agent. Thus, the precipitated "insoluble gel-like" species (nuclei) are mainly solvated by the monomers present in the fluid surrounding the nuclei. Subsequently, the polymerization reaction continues in solution (a), or within the swollen nuclei (b). The polymerization within the nuclei (b) is kinetically favored because the local concentration of monomers in the "swollen individual nuclei” is greater than that in the solution [49]. Moreover, cross-linked or branching polymer chains which may be still forming in solution are "captured" by the growing nuclei, increasing its size. The cross-linked nature of the nucleus prevents them from the mutual penetration and the complete loss of individuality through coalescence. Subsequently, large nuclei are associated with each other in "clusters". This association is conducted through polymer chains that cross-link the nuclei between them. The clusters of polymers remain dispersed in the liquid phase (rich in the porogenic agent) and continue growing. In the last stage of polymerization, the size of clusters is large enough to allow contact with any of its neighbors, forming an interconnected skeleton-type matrix within the system. The interconnected matrix is reinforced through the "inter-globular" growth and the capture of chains still polymerizing in the solution, to obtain finally a porous polymer material. The fraction of the pores into the final macroporous polymer results, after the polymerization reaction, near the volume fraction of the porogenic solvent in the initial mixture of polymerization, since the porogen agent remains trapped into the pores of the cross-linked polymer.

The observation of the factors controlling the pore size in the macroporous polymers is empirical and based on the pore size distributions of macroporous particles of diverse literature. However, the knowledge acquired in the treatment of the particles is not directly applicable to the "rods" evidenced by the fact that these contain large pores or "channels" not commonly found in particle-form polymers. The fundamental difference found in continuous macroporous rods in the distributions of pore sizes is attributed to the polymerization technique used.

An analysis of the suspension systems, in which there are generally two phases (aqueous and organic) reveals that the interfacial tension plays a prominent role in the formation of the drop on the polymerization mixture. This includes control of both size and "spherical" droplet. There is, however, another aspect of the surface tension that is particularly relevant when a suspension polymerization process is conducted for the preparation of macroporous particles: this effect is related to the "shrinkage” occurring during polymerization. The original droplet sizes decrease due to the inevitable shrinkage of the volume. In the early stages of the process of the suspension, the nuclei are dispersed randomly within the droplets (in rotation) in the polymerization mixture. The droplet size decreases due to the shrinkage induced by polymerization, since the interfacial tension exerts a constant pressure on the surface of the drop, retaining the spherical shape; however, the combined effect of interfacial tension and shrinkage produces approximation between the nuclei. Therefore, the interfacial tension contributes to the assembly of dense globules found in the particles.

### Formation of Pores in a Tube during Polymerization

The external conditions of polymerization into a tube are different from those shown in the suspension process. First, a single phase consisting of an organic mixture is present in the tube. The interfacial tension between the aqueous and the organic phase, characteristic of the process of suspension, is absent. Moreover, in contrast to the drops that stay in constant motion in the aqueous phase as a result of agitation, the content of the tube mold remains static during polymerization. The basic mechanism of polymerization in the presence of porogenic solvent described above, including precipitation of the nuclei and shrinkage, is general and independent of the polymerization technique. However, solid nuclei or their clusters have higher density than the polymerization mixture. Thus, in the absence of mixing and if the rate of polymerization is slow, solid nuclei can settle and accumulate in the bottom of the mold (tube). Nuclei (not stirred) and clusters sediment in the bottom of the tube and form a little organized porous structure with large pore volume in the early stages of polymerization. In addition, they present a high specific surface area since the nuclei retain their individuality. Then, as the nuclei come into contact with one another, some growing polymer chains connect the nuclei to each other and the structure is fixed. The polymerization process continues both in solution (above the rod in formation), and within the large pores of the swollen material (formed in the early stages), which leads to the formation of some new nuclei. Thus, the large pores formed initially disappear and the individuality of the polymer nuclei is also lost due to the polymerization reaction that occurs inside the pores and by the capture of the dissolved polymer chains, resulting in a decrease of the surface areas.

In contrast to the "shaped rods", the growing nuclei within the droplets dispersed into the aqueous phase in the suspension polymerization do not lose their individuality. Drops spin by agitation and the centrifugal force allows the nuclei to move into the drop. The nuclei are able to maintain their individuality for a maximum period of time, to grow separately and to package within the particle (drop). As a result, the gaps between the globules forming a single particle are smaller. As mentioned above, the "dynamics of the systems” seems to be the cause of the different pore size distributions between the rods and particles.

The experimental conditions used to obtain porous characteristics (pore size, pore volume and specific surface area) intended for a given polymeric material will depend upon the application field. By varying the reaction parameters in the preparation of these polymers, porous properties can be modified by several orders of magnitude. The parameters that are modified more frequently to achieve this kind of materials are the following: the composition of the porogenic mixture, the reaction temperature, the cross-linking monomer concentration, and the concentration of the radical initiator [48].

The effect of temperature is usually studied since the polymerization temperature can be a variable to consider, adjusting the pore size distribution of the molded rods without requiring a change in the composition of the polymeric mixture. Okay [49] and Svec and Frechet [48,50] concluded that the higher the temperature, the smaller the pores. The polymerization temperature-porous structure relation is a consequence of the increasing decomposition rate of the initiator (and the overall polymerization rate) on increasing the temperature. From these reports, it is known that the higher the reaction temperature, the greater the number of free-radicals generated per unit of time and the greater the number of nuclei and microspheres formed (the amount of monomers in the system is the same for each polymerization). Increasing the number of nuclei and microspheres necessarily decreases their size so that smaller voids or pores between them appear in the final copolymer. In contrast, if the polymerization temperature is low, the rate of polymerization is slow and the transfer of a substantial part of the monomers from the solution in the nuclei can occur, resulting in the growth of the nuclei in a larger size. The pore size distribution is shifted toward greater pores.

On the other hand, as reported by Svec and Frechet [48], the addition of a good solvent to the monolith polymerizing system results in a phase separation that occurs in the “later stages” of polymerization (where cross-linking dominates the phase separation process). Contrarily, the addition of a poorer solvent to the system causes an earlier phase separation in the polymer. The new phase swells with the monomers (if they are liquids) because these are thermodynamically better solvents for the polymer than the porogenic solvent. Therefore, the local concentration of monomers in the swollen gel nuclei is higher than that in the surrounding solution and the polymerization reaction proceeds mainly in these swollen nuclei rather than in the solution. Those nuclei formed are likely to be adsorbed by the large preglobules developed earlier by coalescence of many nuclei, increasing the size of the preglobules. Thus, the globules formed in the system are larger and the voids between them (pores) are larger as well.

If the solvent quality improves, the good solvent competes with monomers in the solvation of nuclei, the local monomer concentration is lower and the globules are smaller. As a result, porous polymers formed in more solvating solvents have smaller pores.

If the monomers are solids and were dissolved in the medium, the effect depends on the solvating power of both monomers and polymer chains in formation [37].

In the case of an increase in the amount of nonsolvating diluent, a decrease is observed in the solvating power of both monomers and polymer chains in formation. Therefore, the nuclei of polymers in formation could segregate and capture preferably monomers from the local solution, and consequently increase their size. This process possibly takes place since the monomers probably tend to pass from a less polar medium to stay within the more polar swollen nuclei, which leads to a higher local concentration of monomers in the swollen nuclei than that in the surrounding solution. The polymerization reaction would proceed mainly in the swollen nuclei rather than in the solution. At the same time, the precipitated microglobules can attract the polymer chains in formation and coalesce with them, which would lead to a further increase in their size and therefore in the size of the pores formed between them. Contrarily, if the solvating power of the solvent increases, the polarity within the nuclei is similar to that in the solution, the local monomer concentration is lower since the monomers are not forced to adsorb preferably into the nuclei and the polymerization occurs with form of nuclei that remain individualized. A large number of nuclei compete for the remaining monomers, leading a higher number of small globules that aggregate with small pores. As concluded previously [6], the thermodynamic conditions that induce phase separation during polymerization depend on the composition of the polymerization mixture containing monomers and the porogen.

The porogenic solvent controls the porous properties of the monolith through the solvation of the polymer chains in the reaction medium during the early stages of polymerization. In view of this, the solvating power of the diluent has a critical effect on the porous structure of monoliths. The addition of a good or a bad solvent produces changes in the phase separation and consequently in the structural heterogeneity [49]. However, it is important to emphasize that high-molecular-weight linear polymers were used to create macropores as inert diluents [6,36,37,49,51,52,53,54]. Synthetic polymers like poly(ethylene glycol) (PEG) could cause lateral chain aggregation leading to the formation of large pores in the preparation of monoliths [6]. Irgum *et al.* [51] demonstrated that the pore dimensions of monolithic materials were improved by varying the molecular weight of linear PEG dissolved in the porogen mixture. They synthesized monoliths from acrylic or methacrylic monomers with a variety of terminal groups with and without ethylene glycol links of differing lengths in the side chains, in combination with triethylene glycol dimethacrylate (TEGDMA) and trimethylol-propane trimethacrylate (TRIM) as cross-linkers.

Alvarez *et al.* [36] prepared macroporous polymer rods from *N*-acryloyl-tris(hydroxymethyl) aminomethane (NAT) and *N,N´-*methylenebisacrylamide (BIS) using different porogenic mixtures and demonstrated that the rod of higher porosity and pore size was formed when dimethylsulfoxide (DMSO) and a 1:1 combination of tetradecanol and PEG 6,000 were used as coporogens. Then, other macroporous polymeric rod systems were prepared by those authors [37,38] using NAT and GMA as mono vinyl monomers and BIS or TRIM as cross-linking agents. The reactions were performed in the presence of a ternary porogenic diluent composed of DMSO, tetradecanol and PEG 6,000. The results showed that a lower polymerization temperature and an increase in the amount of nonsolvating diluents (tetradecanol and PEG 6,000) in the reaction mixture led to products with higher porosity.

On the other hand, porogens such as supercritical carbon dioxide [5], were used to yield structurally controlled porous cross-linked poly(methacrylate) monoliths. Cooper *et al.* [5] demonstrated that it is possible to “fine-tune” the porous morphology in cross-linked poly(TRIM) monoliths by changing the CO_2_ density. In addition, in another research [55] they described the synthesis of porous cross-linked poly(methacrylate) monoliths from radical polymerization using 1,1,1,2-tetrafluoroethane (R134a) as the porogenic solvent. The solvent separation with this porogen was simple and no washing or drying steps were required due to its low boiling point.

Whereas variables such as temperature and porogenic system affect the polymer porosity without changes in the rod composition, the concentration of cross-linking agent affects the porous properties and composition of polymer networks. A decrease in the percentage of the cross-linking agent in the reaction mixture to yield monoliths leads to a shift in the pore size distribution curve toward larger pore sizes [36]. This behavior could be due to the fact that an increase in the cross-linking agent concentration could lead to the formation of more cross-linked nuclei in the early stage of the polymerization reaction allowing an earlier separation phase. The higher cross-linking density of nuclei limits their swelling, and then both monomer diffusion into the nuclei and the real coalescence of nuclei formed in the later stage of the reaction do not occur. Therefore, microglobules have smaller sizes and consequently the voids between them are smaller.

## 4. Hydrodynamic Properties

**Figure 4:**
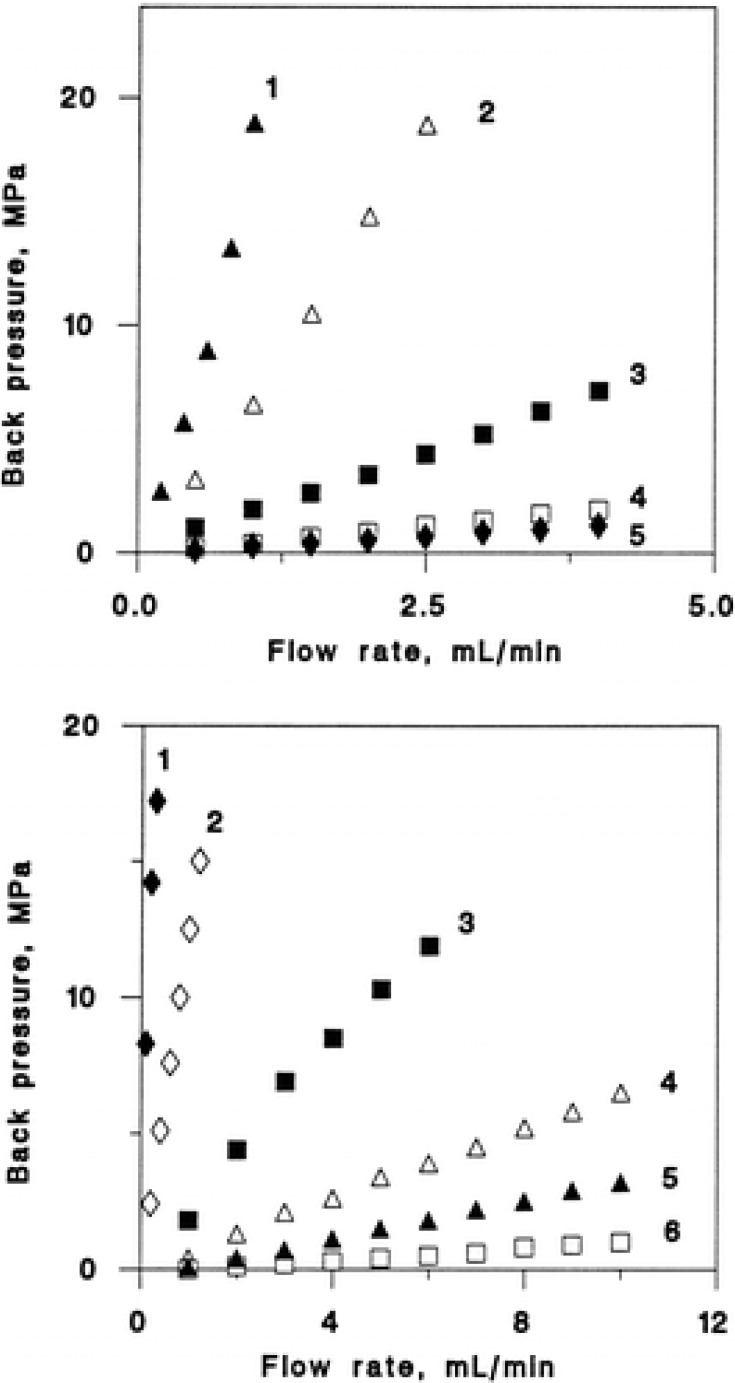
Effect of flow velocity on back pressure in the molded poly(glycidyl methacrylate-co-ethylene dimethacrylate) and poly(styrene-co-divinylbenzene) 100 mm × 8 mm monoliths. Conditions: mobile-phase tetrahydrofuran. (a, top) Polymerization mixture: glycidyl methacrylate 24%, ethylene dimethacrylate 16%, cyclohexanol and dodecanol contents in mixtures 54 + 6%, respectively; temperature 80 °C (1); 54 + 6, 70 °C (2); 54 + 6, 55 °C (3); 57 + 3, 55 °C (4), and 45 + 15%, 70 °C (5). (b, bottom) Polymerization mixture: styrene 20%, divinylbenzene 20%, dodecanol and toluene contents in mixtures 40 + 20%, respectively; temperature 80 °C (1); 40 + 20, 70 °C (2); 45 + 15, 80 °C (3); 45 + 15, 60 °C (4); 50 + 10, 70 °C (5) and 60 + 0%, 70 °C (6). From [3], reprinted with permission from the American Chemical Society.

In the case of separations of biomolecules, the time of residence within the column and the back-pressure are parameters that influence their stability. Regarding the pressure needed to elute the mobile phase, this will be directly related to the size of pores present into polymer rods, requiring low pressure chromatographic supports with large pore sizes. However, a considerable specific surface area (Ss) (characteristic of polymers with low pore size) is necessary to achieve, for instance, greater exposure of functional groups and therefore to yield a good binding capacity with a specific ligand. Thus, the polymer support must have adequate porosity to allow a balance between large pore sizes (to achieve suitable convective transport) and a considerable specific surface area to obtain a good binding capacity. The graph of the pressure required to flow the mobile phase *vs.* the flow rate for polymer rods poly(glycidyl methacrylate-co-ethyleneglycol dimethacrylate) [poly(GMA-co-EGDMA)] [3] is shown in Figure 4 (a, top). As can be seen, high flow rates at relatively low pressures can be achieved. The lines reflect the large pore sizes present in the monolithic media and their rigidity, since at high pressures the media did not contract. If this occurs, the system pressure will increase. In general, media that show these hydrodynamic characteristics are those that contain pores of above 300 nm in size [56].

Figure 4 shows the curve of the system pressure *vs.* flow rates of the mobile phase polymer rods obtained for poly(GMA-co-EGDMA) (a, top) with pore sizes of 100 (1), 470 (2), 1,690 (3), 1,930 (4) and 2,570 (5) nm.

## 5. Chemistry of Polymer Rods

According to the application of the polymer support obtained, a specific chemical functionality in the structure of the polymer rod will be required. For example, continuous polymers formed from hydrophobic monomers can be used as stationary phase in reversed phase chromatography (RPC); those from ionic group-containing monomers could be used in ion exchange chromatography (IEC) or as carriers in capillary electro-chromatography (CEC); and those from chiral units are required for enantioselective chromatographic separations.

**Figure 5:**
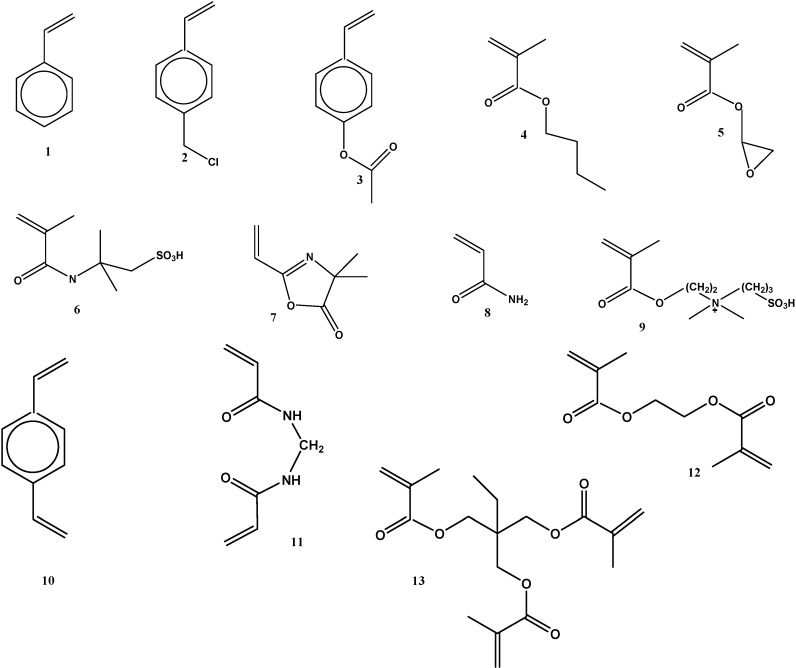
Monovinylic monomers and cross-linkers used in the preparation of porous polymer rods.

As the polymerization mixture consists of a single phase, the possible combination of monomers used in the preparation of continuous polymers is higher than that in the case of suspension polymerization reactions. For this reason, a greater diversity of surface chemical structures could be obtained. Nevertheless, the reaction conditions optimized for a specific system cannot be transferred directly to another system. The reaction conditions for each special system must be optimized. Figure 5 shows different monomers and cross-linking agents used for continuous macroporous polymer synthesis. Hydrophobic monomers such as Sty (**1**) and butylmethacrylate (BMA) (**4**), monomers with reactive functional groups such as the GMA (**5**), chlorometylstyrene (CMS) (**2**) and 2-vinyl-4, 4-dimethylazolactone (VAZ) (**7**), monomers with protected functionality such as 4-acetoxystyrene (**3**), water soluble hydrophilic monomers such as AAm (**8**), acid 2-acrylamide-2-methyl-1-propanesulfonic acid (**6**) and zwitterionic monomer such as *N,N*-dimethyl-*N*-methacryloyloxyethyl-*N*-(3-sulphopropyl ester) ammonium betaine (**9**) were found. The cross-linking agents commonly used are DVB (**10**), BIS (**11**), EGDMA (**12**) and TRIM (**13**).

For several applications, as in the case of affinity chromatography in porous media, the covalent binding of a ligand onto the polymeric material is necessary. The surface functionalization and chemistry and porous properties of the monoliths can be tailored to suit a variety of applications by adjusting the composition of the initial monomer solution and the polymerization conditions [57].

However, on the basis of previous works, the design of successful functionalized monoliths has followed three main strategies: (1) incorporation of monomers that provide functionalities and/or perform post-chemical reactions (activation, modification, *etc.*) on the functional groups of the monolith yielded for the coupling or immobilization of a specific ligand; (2) surface grafting which could greatly increase the number of active sites of the support; and (3) synthesis by Molecularly Imprinted Monoliths (MIP) technique.

### 5.1. Chemical Structure of the Monomers and/or Post-Chemical Derivatization Reactions on the Product

One important factor worth considering is the chemical structure of the monomers used, mainly in relation to the presence of the specific functional groups. The functional groups of the monomers could be used, in a further step of reaction, as anchored groups to immobilize specific ligands or to improve specific properties such as hydrophilicity, hydrophobicity, pH or temperature responsive stimuli. By choosing the correct functional monomer, monoliths for various applications can be designed. For example, two novel anion-exchange polymeric monoliths were prepared by direct copolymerization of 2-(diethylamino)ethyl methacrylate (DEAEM) and poly(ethylene glycol diacrylate) (PEGDA) or copolymerization of 2-(acryloyloxy)ethyl trimethylamonium chloride and PEGDA. The resulting monoliths contained diethyl aminoethyl (DEAE), a weak anion exchanger (WAX) group, and quaternary ammonium (QA), a strong anion exchanger (SAX) group, respectively. Both demonstrated comparable chromatographic properties and did not require additional surface functionalization. The dynamic binding capacities of the two monoliths were similar to or higher than values reported for other monoliths [58].

Buchmeiser *et al.* [59,60,61,62] synthesized macroporous rods through ring-opening metathesis polymerization (ROMP) from bicyclic monomers within borosilicate columns and a ruthenium catalyst, and via the Schrock catalyst triggered ROMP [62]. Buchmeiser *et al.* demonstrated that the performance of norbornene-based monolithic capillary columns prepared via ROMP was improved in separation capabilities for single and double-stranded nucleic acids as well as for proteins [59]. Liu *et al.* reported ring-opening polymerization with synergistic comonomers [63] which contained in a boronate-functionalized polymeric monolith function as a single Wulff-type boronic acid ligand to enable the specific capture of *cis*-diol-containing biomolecules under neutral conditions. In other paper [64], Liu *et al.* reported the preparation of a first-generation boronate functionalized polymeric monolithic column to be used for specific capture of *cis*-diol-containing compounds such as glycoproteins, RNA and carbohydrates.

Jungbauer *et al.* [65] developed affinity monoliths with a different immobilization strategy since the ligand (a peptide) was conjugated to one of the monomers of the polymerization mixture (GMA) prior to polymerization. Thus, after polymerization, a monolithic structure obtained was ready to use for affinity chromatography, for the purification of lysozyme (Lys).

The use of GMA as co-monomer is frequent since it has excellent features due to the presence of epoxy groups which can rapidly react with various reagents. Therefore, the copolymer characteristics can be easily adjusted to the applications desired. Based on poly(GMA-co-DVB) monoliths, DEAE or QA functionalities were introduced to provide WAX or SAX stationary phases for the separation of oligonucleotides, or oligonucleotides and nucleosides, respectively [66,67]. Wei *et al.* [68] presented the preparation of a poly(GMA-co-EGDMA)-based monolith column for weak cation exchange chromatography and its use in the separation of biopolymers. Iminodiacetic acid (IDA)-type adsorbent was prepared by covalent coupling of IDA to the monolithic macroporous poly(GMA-co-EGDMA). Different metal ions (Cu^+2^, Zn^+2^, Ni^+2^) were immobilized on these columns and used for the separation of proteins [69].

Another monomer extensively used in the preparation of monoliths is CMS. Poly(CMS-co-DVB) was synthesized by Geng *et al.* It was chemically modified and tested by separation of biopolymers. The chloro methyl groups of the monolith were used for chemical reaction with ethylenediamine (EDA) and then with chloroacetic acid. A weak cation exchange column was obtained after a two-step modification process [70].

When the immobilization of the ligand through a functional group not provided by the monomers is required, it can be performed by chemical modification reactions on the surface of the material. Sometimes, the epoxy groups of a usual and common monomer such as GMA is hydrolyzed by the formation of a *vic*-diol and oxidized with NaIO_4_ to create aldehyde groups which can be easily coupled to amine-containing ligands as proteins followed by reduction with NaBH_4_ or NaCNBH_3_. Through this, Zou *et al.* [71] immobilized protein A on modified poly(GMA-co-TRIM) and poly(GMA-co-EGDMA) monoliths for affinity chromatography performing the assays on a capillary instrument. The column was used for analysis of human immunoglobulin G (hIgG) in microliter of sample.

Once hydrolyzed the epoxy groups, 1,1´-carbonyldiimidazole (CDI) can be used in an activation step followed by immobilization of antibodies or, as described above, they can be converted to aldehyde groups for the reaction with adipic dihydrazide and NaBH_4_ to create amine groups ready to react with aldehyde-containing materials [72].

The epoxy groups can be ring-opened with secondary amines such as diethyl amine to yield amino hydroxyl-functionalized supports [73,74]. Preinerstorfer *et al.* [75] transformed the epoxy groups of poly(GMA-co-EGDMA) monoliths into 3-mercapto-2-hydroxy-propyl residues via a nucleophilic substitution reaction, using sodium-hydrogen sulfide as nucleophilic reagent.

Poly(CMS-co-DVB) monoliths were submitted to react with EDA to form amine groups ready to couple γ-gluconolactone for HPLC and CEC [76]. Buchmeiser [77,78] and Zou [27] have reviewed various methods of post-modification of polymer-based monoliths.

### 5.2. Surface Grafting

The simple modification procedure only provides a single functionality by reaction of each reactive site of the monolith surface. However, one of the drawbacks of monolithic materials with large pores is the limited surface area of pore walls and the limited amount of functional groups available on the resulting pore wall surface [79,80]. It is known that ligand-containing polymers can be employed for the modification of monolithic supports to increase the ligand density developing a higher binding capacity for chromatographic media. In practice, the final support results more attractive. Thus, polymeric ligands grafted onto a substrate are designed as polymer brushes or tentacles. They would provide multiple functionalities arising from each individual surface site, dramatically increasing the surface group density and providing some special properties [61,81]. Hence, the grafting of solid surfaces with layers of polymers has become a fundamental technique used in areas as varied as microelectronics, packaging, biochips, supports of chromatographic techniques or pH-sensitive membranes [82,83]. In these and other numerous applications, surface grafting enables the introduction of specific properties derived from the grafted layer while preserving the bulk and structural properties of the underlying material. The introduction of functional groups onto the surface of porous monolithic materials via grafting polymerization is a versatile approach for the preparation of materials with controlled incorporation of functional groups [84]. Moreover, active initiating or polymerizable sites located on the surface of the porous matrix were used to carry out the grafting reaction.

Therefore, the primary coverage of the pore surface with the active sites had a decisive effect on the efficiency of the grafting. Changing the concentration of such active sites in the original monoliths is not an easy process and any change in composition during the preparation of the monoliths implies significant changes in their porous structure. In addition, the uses of heat to initiate grafting or increase the initiator concentrations usually improve the functionalization of the entire monolith. Extensive studies performed on different systems demonstrated the complex relation between the nature and the amount of monomers, the reaction conditions and the concomitant porosity [85]. While ingenious solutions were employed to solve the problem, it is clear that each variation in the composition and nature of monomers requires a new search for optimal conditions. This issue became critical in the synthesis and functionalization of monolithic polymers when molecular recognition is a design feature. Therefore, several research works with interesting and innovative techniques have been published about the functionalization of this type of materials.

Direct copolymerization of functional monomers undoubtedly provides the simplest approach for the preparation of functionalized monoliths, and could be another effective approach for introducing ionizable groups onto the surface of monoliths. Using this technique, Gu *et al.* [86,87] designed and synthesized a series of strong cation-exchange monoliths by incorporating different sulfonic acid-functionalized monomers with decreasing hydrophobicity. High-performance separations of peptides and proteins were achieved using these columns. For example, Frechet *et al.* [88] have demonstrated that Ce(IV) initiated grafting poly(2-acrylamido-2-methylpropanesulfonic acid) onto the internal surface of porous monoliths could afford an excellent separation medium for biopolymers and an increase in the dynamic binding capacity.

Anion-exchange polymer chains of poly(2-methacryloyloxy ethyl trimethylammonium chloride) have been grafted onto macroporous polyAAm gels by free radical polymerization. These monoliths achieved protein-binding capacities of up to 6–112 mg/mL, but no separation was demonstrated [89]. Tan *et al.* [90] prepared a polyethyleneimine (PEI) modified ion-exchanger based on poly(methyl methacrylate-co-EGDMA) monoliths. The presence of PEI provided better permeability for the separation of bovine serum albumin (BSA). The binding capacity of the monolithic support was enhanced by increasing the molecular weight of PEI, indicating that the brush ligand derived from the surface captured more protein by multiple binding sites.

DEAE-dextran has been employed by Tan *et al.* [91] to prepare anion-exchange monolithic columns for the separation of proteins. The monolithic column modified with this polymer exhibited an even lower pressure drop and a relative higher binding capacity, which constitute an important potential for a rapid analysis and separation of proteins.

Regarding the preparation of hydrophobic interaction chromatography (HIC) stationary phase for the separation of proteins, the hydrophobicity of separation media can be generally adjusted by selecting the types of ligands. Poly(*N*-isopropylacrylamide) (polyNIPAm) was used as ligand to modify a polymer monolith and the modified product undergo an expected change in hydrophobicity by varying the temperature and/or salt concentration in the process of chromatographic separation. PolyNIPAm is one of most widely studied thermosensitive polymers with low critical solution temperature. This means that the polymers can be subjected to a reversible alteration in their hydrophobic/hydrophilic behavior due to the conformation transition of polyNIPAm chains [92,93]. Svec *et al.* [94] developed a kind of thermal-responsive material. A composite that changes its permeability with temperature leading to the possibility of a thermal gate, a thermal valve, or a thermal control of hydrophobicity/hydrophilicity was reported. A method to modify the internal pore surface of rigid polymer monoliths by grafting-on polyNIPAm is also described. Thus, isocratic HIC of proteins was reported. This constitutes a novel separation technique based on changes in surface polarity (and thus adsorption) accompanying the temperature-induced phase transition of the grafted polyNIPAm. More recently, Yang *et al.* [95] prepared a grafted-polyNIPAm monolith with a controlled amount of polymer ligands. The hydrophobicity of the ligands could be controlled by changing the salt concentration in the chromatographic separation, and sodium sulfate showed the best ability. This monolith enlarged the types of separation media for HIC of proteins, extending this concept.

Photo-grafting can be used for a fast, efficient and versatile surface functionalization. Frechet *et al.* [96,97] showed that the use of simple photo-masks precisely positioned atop of the monolith enables the grafting for proceeding strictly in those confined areas of the monolith exposed to radiation, while no functionalization is observed in dark areas. The photo-grafting of porous three-dimensional materials has been achieved using a benzophenone-initiated surface photo-polymerization within the pores of a macroporous polymer monolith contained in a fused silica capillary.

Irgum *et al.* [3,7] and Frechet and coworkers [98] also reported the successful use of the 2,2,6,6-tetramethylpiperidine-1-oxyl (TEMPO) radical, reversibly trapped during the mold polymerization for grafting the pore surface with functional vinyl monomers *in situ* to easily control the pore surface chemistry.

Wang *et al.* introduced a method for grafting methacrylic acid (MAA) onto the internal surface of hydrolyzed poly(glycidylethylacrylate-co-EGDMA) monoliths with potassium peroxydisulfate in an *in situ* polymerization. The resulting polymer chains showed a pH-responsive monolithic stationary phase used for the fast purification of the Lys from chicken egg white (CEW) [99].

On the other hand, several advantages can be reached when ROMP-based route is used. Functionalized continuous rods were synthesized by one additional synthetic step that takes advantage of the living character of the ROMP-based copolymerization [100]. This “*in situ*” derivatization was achieved after the formation of the continuous rod by reacting the active, surface bound initiator with functional, norborn-2-ene and 7-oxanorborn-2-ene-based monomers. The ROMP-based route permited the preparation of a great variety of monolithic stationary phases for liquid chromatography since the functionality tolerance of these polymerization techniques allows for creating chromatography supports with an unprecedent diversity in terms of functional groups that may be introduced [101,102]. Buchmeiser *et al.* described ring-opening metathesis polymerization- (ROMP-) based monoliths that were synthesized using Grubbs`first generation catalysts. In these protocols, the surface-immobilization was realized by grafting techniques. Afterwards, the final products were used in metathesis-based reactions including ring-closing metathesis (RCM), ring opening cross metathesis and enyne metathesis [103,104,105]. Finaly, applying Grubb´s first generation benzylidene-type catalyst in ROMP of norborn-2-ene and 1,4,5,8,8a-hexahydro-1,4,5,8,exo,endo-dimethanonaphtalene, various monoliths were prepared for the micropreparative separation of DNA fragments [106].

### 5.3. Synthesis of Molecularly Imprinted Monoliths

Molecular imprinting is a useful technology to create recognition sites in a macromolecular matrix using a template molecule [107]. The molecular imprinting technique needs a synthetically prepared polymer matrix apt to form cavities sculpted around the template molecules of the same kind [98]. Molecularly imprinted polymers (MIPs) are easy to prepare, stable, inexpensive and capable of molecular recognition. They have been used in various analytical separation domains such as liquid chromatography [109,110], capillary electrophoresis [111,112] or sensors [113,114], however, the most intensively studied application is that in solid-phase extraction (SPE) media [115,116,117]. Therefore, MIPs can be considered as artificial affinity media.

*In situ* polymerization is a vey simple and easy method for preparing MIPs for HPLC or SPE separation. In a typical process, the reaction mixture containing the molecule (mold or template), functional monomers, a high concentration of cross-linking agents, porogenic agents and initiators are poured into a stainless-steel tube, sealed at one end, and degassed ultrasonically. Then, the other end is sealed and the polymerization reaction is allowed to process by heating. Once polymerization occurs, the molecule used as mold is removed by washing from the polymeric structure, remaining "*printed*" with the shape of the template molecule. After the removal of the template, the column of MIP monolith can be connected directly to the HPLC system for online SPE or analysis of target molecules [118].

Recently, some efforts have been made to prepare monolithic MIPs in molds, e.g. capillaries, since no crushing, sieving and packing are necessary [119]. Liu and coworkers [120] prepared a MIP monolith by *in situ* polymerization using Sty, GMA and MAA as monomers, DVB and triallyl isocyanaurate as cross-linking agents, and a ceramide as template molecule. The results showed that using ceramide as template molecule significantly affected the pore structure and pore distribution of the monolith, and greatly improved the retention of ceramide and its analogues used in cosmetics as well. This indicated the potential of ceramide imprinted monolith synthesized in the online SPE of ceramides from yeast.

Denizli *et al.* [121] studied and assessed the Fe(III)-imprinted poly(HEMA-*N*-methacryloyl-(*L*) cysteine methyl ester) monolith for its capability to remove Fe(III) in vitro from human plasma with β thalassemia. Remcho and coworker [122] reported the preparation of novel monolithic columns with porosity determined by spherical particles of silica. This approach erradicated one of the variables involved in the design of polymer composition: the need for a porogenic solvent. In addition, altering the surface characteristics of the templating beads influenced the composition of the finished monolith surface. For example, the results demonstrated that octadecyl modified silica particles interacted with the hydrophobic moieties of monomers before the initiation of polymerization, thus specifing their orientation in the resulting polymer.

MIPs present the ability to selectively discriminate between very similar molecules, which can be applied in the resolution of racemates and in selective catalytic processes. Sinomenine (SIN), an alkaloid with analgesic and antiinflammatory effects, was used as template for the synthesis of an imprinted polymer with specific recognition ability for SIN. It was prepared by *in situ* molecularly imprinted technique by Fu *et al.* [123]. This study has shown that SIN-imprinted copolymers of poly(MAA-co-EGDMA) provide efficient extraction of SIN from herb matrices and rabbit blood. MIPs were used with poly(acrylic acid-co-EGDMA) [poly(AAc-co-EGDMA)] rods to resolve positional isomers of different diamine naphtalenes and enantiomers of anilides of the phenylalanine [124]. Matsui *et al.* used the same support for the resolution of various alkaloids of cinchona [125].

**Figure 6:**
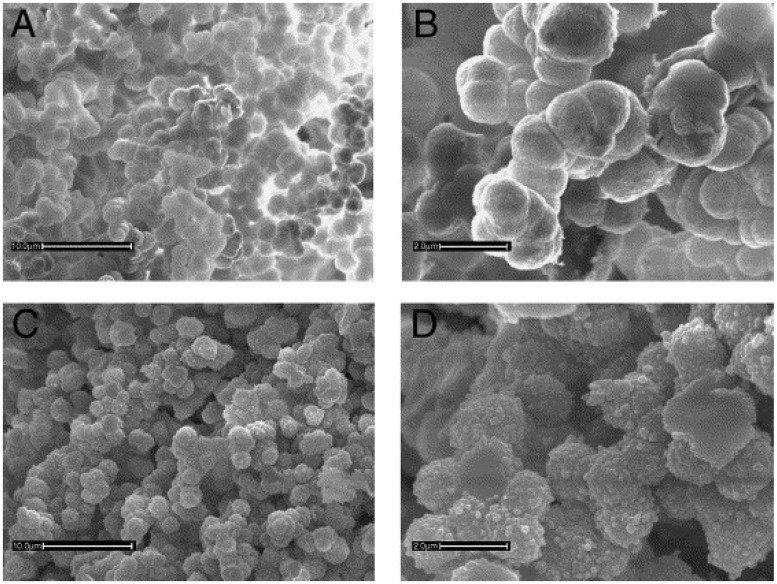
Scanning electron micrographs of (A) nongrafted core monolith at magnification 3,000×; (B) nongrafted core monolith at magnification 10,000×; (C) grafted BV-mMIP at magnification 3,000× and (D) grafted BV-mMIP at magnification 10,000× [126]. Reprinted with permission from Elsevier.

The molecular imprinting of polymer technique is also applied to the surface of monoliths. Therefore, it is interesting to use this technique during the grafting of the monolith to obtain an even better molecular recognition with binding sites that will not be encapsulated in the bulk, but will be directly present on the surface material providing a faster access to the analyte. Irgum *et al.* [126] reported MIP grafted monoliths using a TRIM core material photo-polymerized *in situ* in a 100 µm I.D.UV-transparent capillary and further photo-grafted to create specific cavities in the grafted layer for sample enrichment. These separation media have been evaluated by means of HPLC for their capabilities to recognize three local anesthetic compounds with close structural similarity. The retention factors were determined and compared with the nonimprinted reference column, yielding high imprinting and good selectivity factors between the three analytes. According to the authors, a high percentage of TRIM in the polymerization mixture ensures that a large number of free methacrylate terminal groups are available on the pore surfaces to act as anchoring points for the MIP grafting. This polymerization technique allowed imprints to be directly created on the surface of the polymer reducing the amount of template used for their preparation. The surface morphology of macroporous polymers with and without the grafting layer can be seen in Figure 6.

Different mMIPs were synthesized using bupivacaine, mepivacaine and *S*-ropivacaine molecules as model templates. The retention properties and cross-selectivity of the materials were tested on a microsystem against each analyte obtaining materials with high imprinting and good selectivity factors between the three. A study with a pure enantiomeric target (*S*-ropivacaine) was also carried out showing very high retention for the analyte itself.

## 6. Important Applications

Because of their porous characteristics and easy preparation, continuous porous polymers with a considerable specific surface area and large pore size and pore volume, have been widely used in processes which required high flow rates and low pressures. The more important application of monoliths is that as stationary phases in HPLC. This and other useful fields of application are detailed in the sections that follow: use as stationary phase in HPLC; use as reactive supports; use as bioreactors; use in detection in solid-phase; use in solid-phase extraction and decontamination.

### 6.1. Continuous Porous Polymers as Stationary Phases in HPLC

As mentioned above, the particle-form supports used as stationary phases are particularly inefficient when used in the separation of high-molecular-weight molecules, such as synthetic polymers or biological molecules, since they have diffusion coefficients by several orders of magnitude smaller than those of the low-molecular-weight solutes. This problem led to the preparation of continuous porous polymers, which usually have a high binding capacity and large pores that allow performing chromatographic separations of high-molecular-weight molecules in a few minutes. For this reason, one of the most important applications of monoliths is that as supports in different chromatography processes where polymers are prepared into either HPLC columns or fused silica capillaries. In this review, we are reporting research on the use of macroporous polymer rods in different chromatographic processes. For detailed information on this application, Vlakh and Tennikova [127] have recently pubished a comprehensive review on this subject.

**Figure 7:**
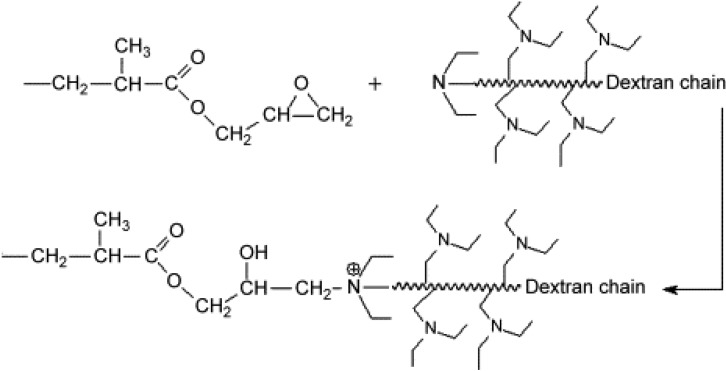
The illustration of reaction between DEAE-dextran and monolithic media [91]. Reprinted with permission from Elsevier.

Tan *et al.* [91] reported the use of poly(methylmethacrylate-co-EGDMA) monolithic polymers modified with DEAE-dextran in the separation of proteins through the anion-exchange mode. The preparation of these supports is shown in Figure 7. Fast separation of Lys, hemoglobin and BSA on the column was achieved with these polymers within 3 min at flow rates of 1,445 cm/h.

**Figure 8:**
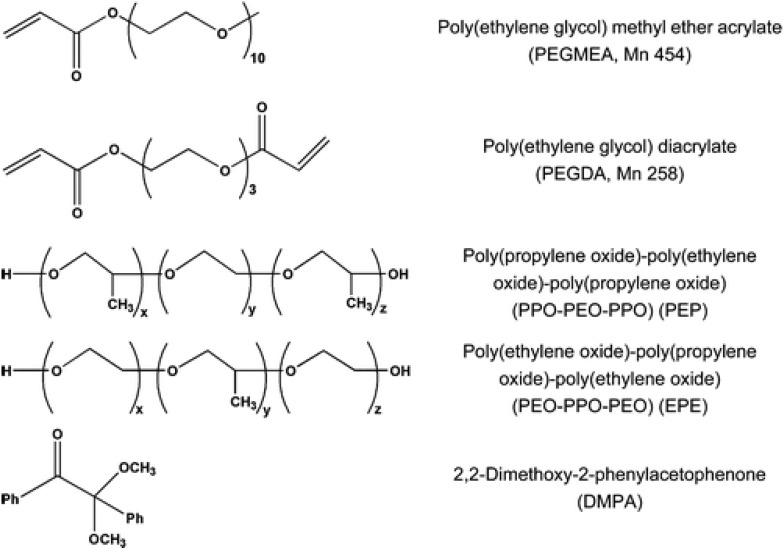
Structural formulas of reagents for synthesizing the monoliths [128]. Reprinted with permission from the American Chemical Society.

In a recent report, porous monoliths have been used as supports in the separation of proteins and peptides by size exclusion chromatography (SEC). Li *et al.* [128] synthesized porous monoliths in fused silica capillary columns by photo-initiated polymerization and evaluated them in SEC separation of proteins and peptides. The monomers employed were poly(ethylene glycol methyl ether acrylate) (PEGMEA) and PEGDA as mono vinyl monomer and cross-linker, respectively. The originality of this work lies in the use of mixtures of ethyl ether and nonionic surfactants poly(propylene glycol)-block-poly(ethylene glycol)-block-poly(propylene glycol) (PPO-PEO-PPO, abbreviated as PEP) or PEO-PPO-PEO (abbreviated as EPE) as porogenic solvents. The nonionic surfactants were used to create a considerable portion of mesopores for size exclusion of proteins. The structures of the different compounds used in the preparation of the monoliths are shown in Figure 8. Using these polymers, the separation of proteins and peptides was achieved efficiently.

Isobe and Kawakami [129] reported the preparation of a convection interaction media (trade name CIM^®^, BIA Separation, Ljubljana, Slovenia), an isobutyl monolithic disc to the purification of the primary alcohol oxidase (from *Aspergillus ochraceus*) by HIC. The discs were synthesized through the reaction between the CIM epoxy disc and isobutylamine. The authors showed that the enzyme was adsorbed on this column and eluted with high purity. In another study [130] the authors reported the use of long alkyl chain methacrylate monolithic polymers as stationary phases in reverse phase liquid chromatography (RPLC). The monoliths were prepared in 100 μm i.d. capillaries by a radical copolymerization of stearyl methacrylate (SMA) with EGDMA with a mixture of isoamyl alcohol and 1,4-butanediol as porogenic solvent. The supports were tested in the separation of mixtures of weak acids (phenols compounds), neutral (polycyclic aromatic hydrocarbons) and basic compounds (anilines) showing a good chromatographic performance.

**Figure 9:**
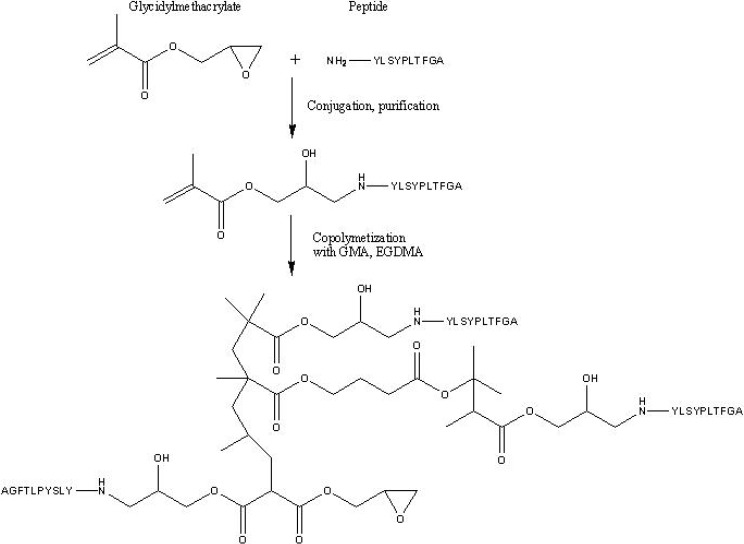
Schematic drawing of the preparation of an affinity monolith produced by *in situ* polymerization [131]. Reprinted with permission from the American Chemical Society.

Affinity chromatography is one of the chromatographic processes in which macroporous monoliths are most used. Thus, protein A was immobilized in a monolithic capillary column using GMA as monomer and TRIM and EGDMA as cross-linkers, respectively. The capillary affinity column obtained has been successfully applied for the rapid separation of hIgG in human serum. Hahn *et al.* [131] developed an affinity monolithic support with a novel immobilization strategy which consisted in the *in situ* polymerization of the ligand. In this study, the model ligand (a peptide directed against Lys) was reacted to the GMA monomer prior to polymerization. Afterwards, with the conjugate, GMA and EGDMA, a monolith was formed and tested against Lys. The results showed a higher affinity constant compared with that obtained with conventional sorbent. The different steps used in the preparation of this affinity support are illustrated in Figure 9.

Porous poly(GMA-co-EGDMA) monoliths were assayed as a stationary phase in dye-affinity chromatography using Cibacron Blue as ligand [132]. The supports were employed in the adsorption/desorption of BSA from aqueous solutions and human plasma. The supports assayed presented a very low and nonspecific adsorption of BSA (0.8 mg/g) and an adsorption amount of BSA from human plasma of 53.2 mg/g with a purity of 92%.

In addition, macroporous monolithic rods were used in immobilized metal affinity chromatography (IMAC) for the purification of histidine-tagged lentiviral vectors [133] and the separation of proteins [69]. In both cases, IDA was employed as the metal chelating ligand. In the latter work, Luo *et al.* optimized the coupling of IDA onto the epoxide groups present in the poly(GMA-co-EGDMA) matrix through the study of the influence of time and temperature reaction. Temperatures between 70–80 °C and 16 h were the most favorable conditions. By employing these supports, the purification of Lys from egg white and BSA was successfully performed.

### 6.2. Continuous Porous Polymers as Reactive Supports

A high chemical reactivity and a large capacity of accessible functional groups are the requirements for the application of supports in solid phase chemistry. Within this field, continuous poly (CMS-co-DVB) was used as acylating resin to transform amines into amides [134]. To obtain these resins, the radical initiator 4,4'-azobis(4-cyanovaleric acid) was attached to the surface of the resin. Subsequently, grafting reactions were performed with acetoxystyrene or CMS. Acylation reactions were carried out by passing the reactant mixture through the resin in different solvents. It should be noted that these polymers could be easily regenerated and reused.

Kunz *et al.* [135] developed composite materials consisting of inorganic carrier materials with monolithic polymers incorporated in their structures which were used as supports to carry out different chemical reactions in a flow-through mode. Therefore, carrier materials (with pores in the micrometer up to millimeter range) were immersed in the polymerization mixture used for the synthesis of the macroporous monoliths, and the polymerization reactions were started by radical polymerization. Thus, by introduction of the inorganic support, a second monolithic material was created. The reaction conditions were chosen in such a way that pores with diameters in the micrometer range were formed. Thus, the final composites were highly porous materials, allowing good penetration of liquids at low-pressure drop. The large number of monolithic materials synthesized with different polymer functionalities was used in a wide variety of chemical reactions such as selective reductions, Suzuki cross-coupling reactions, nucleophilic substitutions, and catalytic transfer hydrogenations, obtaining very high yields in all cases.

**Figure 10:**
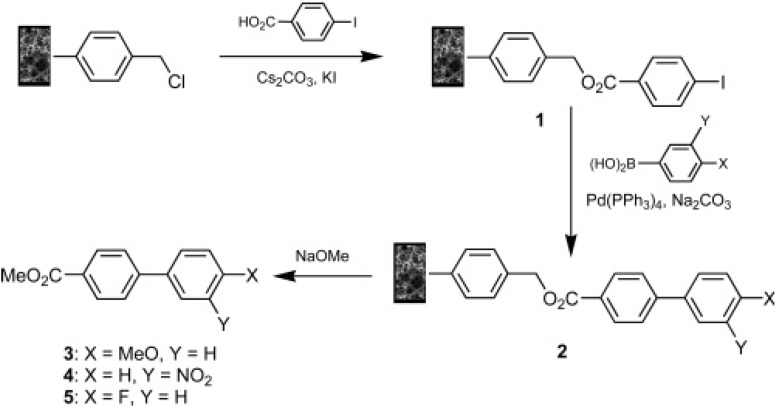
PolyHIPE-supported synthesis of biphenyl-4-carboxylic acid methyl esters [136]. Reprinted with permission from the American Chemical Society.

Brown *et al.* synthesized “high internal phase emulsion polymer” (polyHIPE) monoliths to be used as polymer-support for Suzuki cross-coupling reactions (Figure 10) [136]. PolyHIPEs containing high loadings of chloromethyl groups were efficiently prepared by the direct copolymerization of 4-vinylbenzyl chloride and DVB monomers. The supports were used in batch and flow-through modes and a high yield of a pure biaryl product was obtained using the polyHIPE support in cubic form and an electron-rich boronic acid.

### 6.3. Continuous Porous Polymers as Bioreactors

The immobilization of enzymes on solid supports is of particular interest in biocatalytic processes. Some of the advantages offered by these materials include the ease of separation of the catalyst support upon completion of the reaction and the possibility of reuse in several reaction cycles.

**Figure 11:**
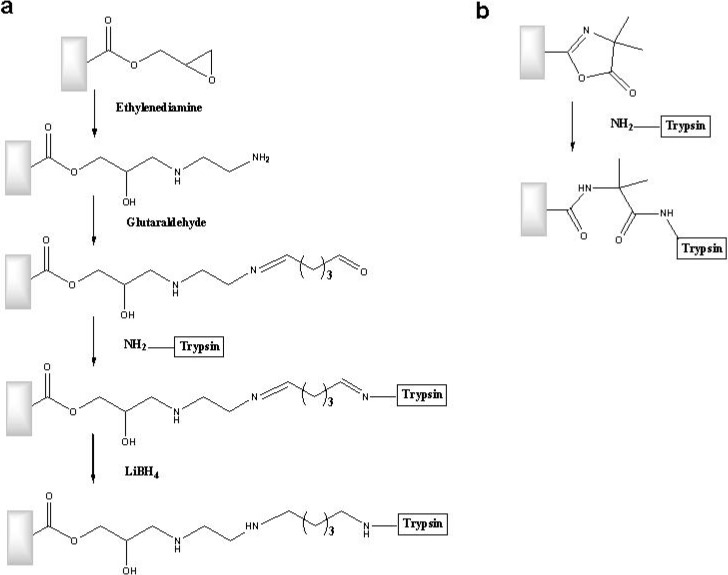
Sequence of reactions in immobilizing trypsin on rods of (a) poly(GMA-co-EGDMA) and (b) poly(VAZ-co-AAm-co-EGDMA). Adapted from references [137,138].

In one of his works, Petro *et al.* reported comparative studies using trypsin immobilized on poly(GMA-co-EGDMA) in particle-form and monolithic rods [137]. The sequence of reactions for immobilizing the enzyme is shown in Figure 11(a). The results indicated that the enzyme immobilized in the rod-shaped support presented higher activity than that in the particulate-shaped support at a linear rate of 25 cm/min. These differences were due to the increase in the mass transfer rate as a result of convective transport reached with the rod-shaped support. In addition, the monolithic bioreactor showed activity at flow rates of even 40 cm/min, whereas the particulate support considerably increased the pressure of the system at these flows rates. In another paper [138] it was reported the immobilization of trypsin on substrates of poly(VAZ-co-AAm-co-EGDMA). The immobilization reaction is shown in Figure 11(b). The polymer proved to be more hydrophilic and more suitable for processes involving biological molecules. The coupling of the enzyme was facilitated by a single step.

In another research reported by Svec, Fréchet and coworkers, the azlactone functionalities were used for the preparation of enzymatic microreactors synthesized into capillaries and microfluidic chips [139]. The porous monoliths consisted of VAZ, EGDMA, and AAm or HEMA were prepared by UV-initiated polymerization reaction at room temperature. Once the macroporous polymer was obtained, the trypsin immobilization was carried out using a simple mechanical pump demonstrating the open-pore structure of the supports. The proteolytic activity of the enzymatic microreactor on chip was demonstrated using a fluorescently labeled casein and myoglobin as substrates at a very fast flow rate which afforded very short digestion time. The digest was then characterized using MALDI-TOF MS and sequence coverage of 67% was reached.

**Figure 12:**
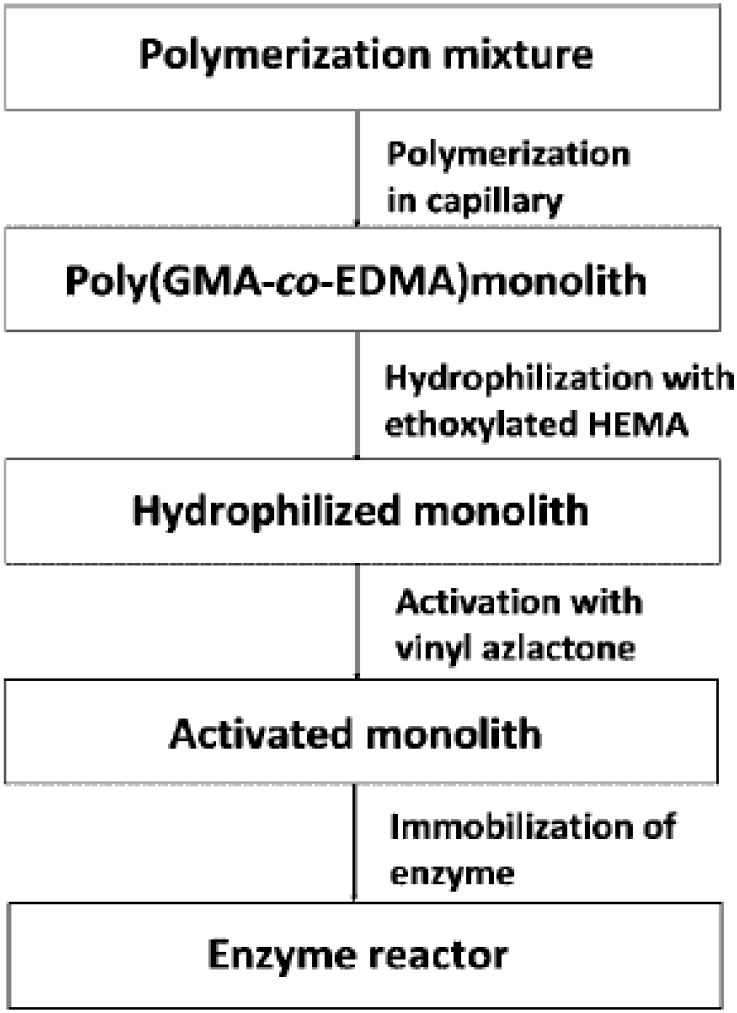
Scheme of the preparation of the optimized monolithic support for the immobilization of proteolytic enzymes [140]. Reprinted with permission from the American Chemical Society.

Sometimes when hydrophobic proteins are analyzed, a proper hydrophylyzation of the polymer surface is required to avoid hydrophobic nonspecific interactions. Therefore, a novel approach was recently developed for the modification of the surface chemistry of macroporous poly(GMA-co-EGDMA) via multistep/multilayer photo-grafting reactions to obtain capillary enzymatic microreactors containing trypsin and endoproteinase LysC for the characterization and identification of proteins [140]. First, the monolith was hydrophylyzed via photo-grafting of poly(ethylene glycol methacrylate) followed by photo-grafting of a VAZ to provide a polymeric surface with the functionalities required for immobilization. This new approach minimized the undesired nonspecific adsorption of proteins and peptides and facilitated the control of enzyme immobilization and protein digestion processes. Figure 12 shows the different reaction steps followed in the synthesis of these enzymatic reactors.

Once the conditions for the digestion were optimized, the bioreactors were then coupled into a multidimensional system comprised of a monolithic capillary enzyme reactor, an inline nanoLC separation of peptides using a poly(lauryl methacrylate-co-EGDMA) monolithic column, and ESI/TOF MS. With this system, the digestion of high-molecular-weight human IgG was carried out at room temperature attaining in a few minutes a similar digestion extent with soluble enzyme at 37 °C after 24 h. Therefore, these results demonstrate the advantages of the monolithic bioreactors in terms of reaction time and temperature.

In a recent report, Loos and coworkers [141] have modified the copolymer poly(GMA-co-EGDMA) with five different amines applied to *Candida Antarctica* lipase B immobilization. The activity of the immobilized ligand was significantly improved showing a higher activity than that of the free enzyme.

### 6.4. Continuous Porous Polymers in Detection in Solid-Phase

The chemiluminescence of peroxy-oxalate is used as a very sensitive method for detecting hydrogen peroxide. Generally, these experiments were performed with carriers in particulate form, containing a fluorophore attached to their surface. Ponten *et al.* carried out photo-polymerization to reach poly(GMA-co-TRIM) rods [142]. The supports were modified with 3-aminofluoroantene and were evaluated in the detection of hydrogen peroxide proving to be highly efficient with respect to the generation of light.

On the other hand, Sherrington *et al.* developed an optical sensor using anisotropic imprinted polymer rods [143]. They prepared a continuous transparent polymer in the presence of a photo-labile mold molecule and then irradiated the polymer with plane-polarized light. According to the orientation of their dipole moments, the template molecules were able to absorb polarized light converting them into reactive species, able to be inserted in the polymer chains. A subsequent extraction of the unreacted mold molecules allowed obtaining an anisotropic material that contained cavities with the template molecular-shape and with a particular orientation able to recognize it in the presence of other structurally similar molecules. The presence of the mold molecule in the mixture could alter the degree of anisotropy of the polymer, measurable by spectroscopic techniques.

### 6.5. Continuous Porous Polymers in Solid-Phase Extraction

Polymers used as supports for solid phase extraction must have good hydrodynamic properties and an important specific surface. As mentioned above, the continuous porous polymers present these requirements, which convert them in potential carriers for the application in this field. Xie *et al.* [144] prepared Sty-derived polymer rods with specific surface as high as 400 m^2^/g, being permeable to liquid at reasonably low pressures. These polymers were used as supports in SPE of different phenols at high flow rates. These phenols were adsorbed 30 times more than conventional media.

**Figure 13:**
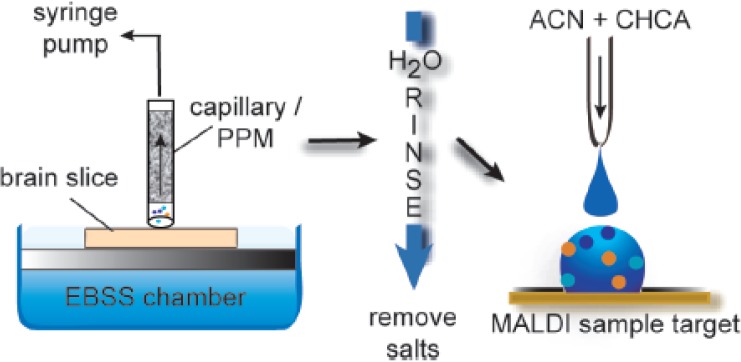
Scheme showing the sampling process from a mouse brain slice using a capillary loaded with PPM; the individual steps shown include peptide collection, a monolith rinse and peptide deposition onto the matrix-assisted laser desorption/ionization (MALDI) target for MS characterization [145]. Reprinted with permission from the American Chemical Society.

In a recent work reported by Iannacone [145], monolithic columns were employed to collect and concentrate neuronal release from invertebrate and vertebrate model systems, prior to their characterization with mass spectrometry. The supports were prepared in fused-silica capillaries from lauryl methacrylate (LMA) and EGDMA. Their binding capacities (determined using fluorescein and fluorescently labeled peptides) were on the order of nanomoles per millimeter of length of monolith and their limit of detection (determined for angiotensin I and insulin in artificial seawater) was on the order of femtomole. Then, to evaluate their behavior for collecting and concentrating peptide release, the capillaries were positioned directly above, but not in contact with the surface of the bag cell cluster from the *A. californica* abdominal ganglion, as well as cortical regions of a mouse coronal brain slice (see Figure 13). Upon electrical or chemical stimulation, the secreted chemicals were retained on the monolith, washed of excess salts, released from the porous polymer, and detected with MALDI-TOF MS. Comparing these results with those obtained using individual beads placed on brain slices, the monolithic capillaries showed greater binding capacity and maintained higher spatial resolution, compared to the larger-volume, solid-phase extraction collection strategies.

**Figure 14:**
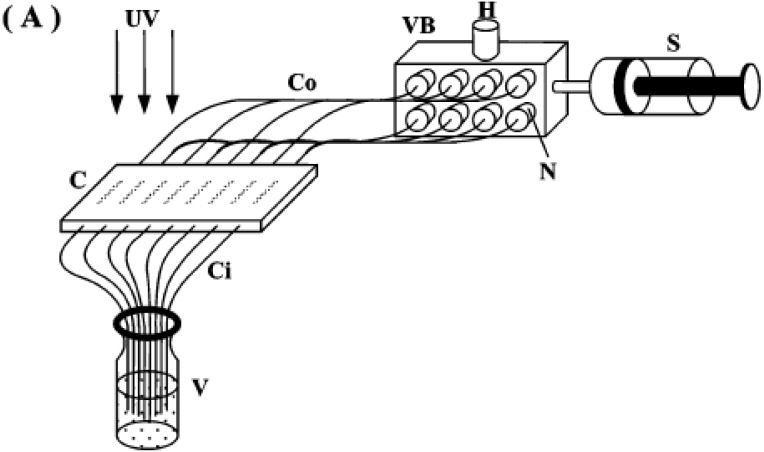
Experimental setup for the polymerization preparation of monolith: S, 10-mL plastic syringe; N, nanoports; VB, vacuum distribution box; H, vent hole; C, polymer substrate chip; Ci, Co, inlet/outlet connecting capillaries; UV, UV light; V, vial containing monomer mixture [146]. Reprinted with permission from the American Chemical Society.

In another interesting approach to the development of microfludic chips to be used as supports in SPE, Tan *et al.* [146] prepared an array of eight porous monolithic columns into a polymeric chip to be tested for SPE cleanup of biological samples prior to directly coupled electrospray mass spectrometry (ESI-MS). The polymers were synthesized using BMA and EGDMA as monomers. The polymerization mixture was vacuumed simultaneously into the eight parallel channels (10 mm long, 360 *í*m i.d.) using a homemade vacuum manifold device and polymerized in parallel under UV irradiation. The experimental setups for the polymers preparation are shown in Figure 14.

The different binding parameters were evaluated using imipramine as a pharmaceutical test compound obtaining high recovery and good reproducibility. To demonstrate the analytical potential of the chip-based SPE system with real-world samples, human urine and P450 drug metabolism incubation mixture samples were tested. The results suggest that the chip-based monolithic columns are applicable to SPE cleanup of real-world biological samples. Although in this work the authors used an array of eight SPE columns on a polymer-based substrate, they anticipated that an array of 96 (8 × 12) or 384 (16 × 24) monolithic columns in a polymer device could be developed and used for a larger number of samples.

### 6.6. Continuous Porous Polymers in Decontamination

**Figure 15:**
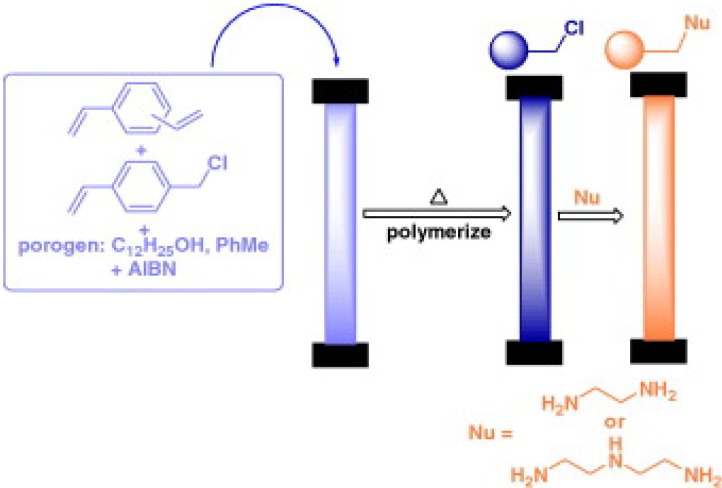
Preparation and modification of monolithic columns [147]. Reprinted with permission from Elsevier.

The monolithic polymers present a permanent porous architecture which allows their use in the clean-up of the fluorinated solvents. Korotchenko and Gagné [147] developed porous organic polymer columns for the flow-through nondestructive removal of a ‘‘mustard gas” simulant 2-chloroethyl phenyl sulfide (CEPS), from the fluorinated solvent HFE-7100 (a mixture of methyl nonafluorobutyl ether and methyl nonafluoroisobutyl ether). First, the monolithic polymers were synthesized by the *in situ* copolymerization of DVB and CMS in stainless steel tubes followed by a derivatization treatment with EDA or diethylenetriamine. Figure 15 shows the synthesis and functionalization of the monolithic supports.

Once the polymers had been functionalized, the decontamination of CEPS was carried out through nucleophilic substitution reactions between the CEPS molecule and the nucleophilic polyamino groups on the polymeric surface. The results obtained indicated that polyamine-modified supports had a high affinity for CEPS, with removal efficiencies of up to 97%.

## 7. Concluding Remarks

Macroporous monolithic materials in rod form have been introduced as useful generation of polymers to be tested and applied in different fields. They may be prepared in a more simple way respect to suspension from a homogenous mixture *in situ*, into a mold. These materials contain large interconnected pores or channels allowing for high flow rates at moderate pressures. The open pore structure of the monoliths provides the high accessibility for large biomolecules and high-molecular-mass compounds in general. Extensive evaluations have documented and explained how their unique architecture creates unique and important fractionation characteristics. The great potential of these separation media was so confirmed.

In this review, we have outlined the developments in the preparation of these materials. It has been demonstrated that a variety of porous structures can be obtained during crosslinking processes by changing the variables of the rod synthesis, *i.e.*, the diluents and the temperature as well as the monomers amounts or concentration. Besides, we presented important reports from contributions of several groups working in the design of these macroporous monoliths. So, we summarized the design of successful functionalized monoliths by three main strategies: (1) incorporation of monomers that provide functionalities and/or performing post-chemical reactions on the functional groups; (2) surface grafting which could greatly increase the number of active sites of the support and (3) synthesis by Molecularly Imprinted Monoliths (MIP) technique. Different useful fields of application of such polymers as: stationary phase in HPLC; reactive supports; bioreactors; use in detection in solid-phase and solid-phase extraction; and decontamination, were considered for these materials.
